# Efficacy of sequential therapies with sorafenib-sunitinib versus sunitinib-sorafenib in metastatic renal cell carcinoma: A systematic review and meta-analysis

**DOI:** 10.18632/oncotarget.14671

**Published:** 2017-01-15

**Authors:** Tingyu Wen, Hai Xiao, Chao Luo, Li Huang, Meimei Xiong

**Affiliations:** ^1^ College of Basic Medical Sciences, Gannan Medical University, Ganzhou, China; ^2^ Department of Pathology, Gannan Medical University, Ganzhou, China; ^3^ Department of Urology, People’s Hosptial of Pingxiang, Pingxiang, China; ^4^ Department of Oncology, The First Affiliated Hospital of Gannan Medical University, Ganzhou, China; ^5^ Department of Nephrology, The First Affiliated Hospital of Gannan Medical University, Ganzhou, China

**Keywords:** renal cell carcinoma, sorafenib, sunitinib, targeted agents, meta-analysis

## Abstract

The most efficient sequence of targeted agents for metastatic renal cell carcinoma patients has yet to be identified. Whether the sequence of sorafenib and sunitinib really matters is controversial and not answered clearly until now. This meta-analysis aims to estimate the efficacy of receptor tyrosine kinase inhibitors sorafenib-sunitinib and sunitinib-sorafenib for metastatic renal cell carcinoma, on the outcome of first-line progression-free survival, second-line progression-free survival, total progression-free survival and overall survival.

We searched PubMed, Embase, Cochrane Library and ClinicalTrails.gov for eligible studies. Data were analyzed using random or fixed effects model depending on the heterogeneity of the eligible studies. Heterogeneity across studies were analyzed using Q and I2 statistics.

Of 902 identified studies, ten were qualified in our analysis (*N* = 1732 patients). Sorafenib-sunitinib yielded no statistically significant benefit in first-line progression-free survival (fixed effects; HR = 0.95; 95%CI 0.75-1.21; *p* = 0.702), total progression-free survival (random effects; HR = 0.92; 95%CI 0.71-1.19; *p* = 0.531) and overall survival (fixed effects; HR = 0.89; 95%CI 0.72-1.09; *p* = 0.257), compared with sunitinib-sorafenib. Second-line progression-free survival was longer for sorafenib-sunitinib than sunitinib-sorafenib (fixed effects; HR = 0.55; 95%CI 0.44-0.68; *p* = 0.000).

Sequential therapies with sorafenib and sunitinib is well tolerated and efficient in mRCC. However, there are no evidence supported that sorafenib–sunitinib has the superiority to sunitinib-sorafenib in sequence. The ideal sequence of targeted agents requires further elucidation.

## INTRODUCTION

Metastatic renal cell carcinoma (mRCC) is the spread of the primary renal cell carcinoma from the kidney to other organ [1]. Due to its mild clinical signs, as many as 30% of people have metastatic disease by the time they are diagnosed with renal cell carcinoma [2]. The most common sites for metastasis are the lymph nodes, lungs, bones, liver and brain [3]. mRCC is notoriously resistant to available chemotherapy and radiotherapy [4], and the 5 year survival rate for mRCC remains under 15% [5].

Nowadays, the National Comprehensive Cancer Network Kidney Cancer Panel (NCCN, Version 2.2017) recommends sunitinib and sorafenib as preferred category 1 and category 2A options for first-line treatment of patients with relapsed or medically unresectable predominantly clear cell stage IV renal carcinoma, respectively [6]. Moreover, sunitinib and sorafenib has also been listed as category 2A subsequent therapy options [6].

Sequential therapies with distinct targeted drugs have become a standard protocol when patients suffering from disease advanced during treatment [7]. Consequently, probing how to sequence molecular targeted drugs in an optimal way is essential for maximization of clinical benefit in patients with mRCC. Does the therapeutic order of sorafenib and sunitinib in mRCC really matter? Parts of recent retrospective studies have suggested that sorafenib followed by sunitinib (So-Su) was optimal to sunitinib followed by sorafenib (Su-So) in progression-free survival and overall survival [8–12]. Nevertheless, a recent randomized controlled trial SWITCH [13] and several retrospective studies [14–17] argued that So-Su and Su-So offer similar clinical efficacy in mRCC, with no statistically differences.

Recognizing the divergent findings and that a single study might not be able to provide sufficient evidence into clinical recommendation. We therefore produced this systematic review and meta-analysis to evaluate the efficacy of So-Su versus Su-So on the outcome of first-line progression-free survival, second-line progression-free survival, total progression-free survival and overall survival.

## RESULTS

### Search results

**Figure 1:**
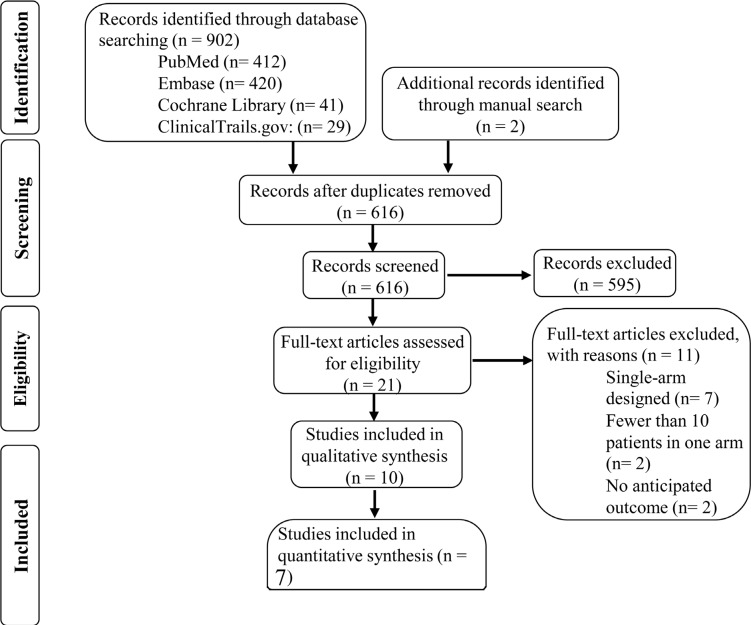
Study selection process

The search of PubMed, Embase, Cochrane Library and ClinicalTrails.gov provided 902 recordings total. In addition, manual search revealed 2 additional records. Then, 21 were selected for full-text review depending on pre-planned inclusion criteria. Finally, 7 studies were available for quantity analysis owing to their adequate quality and sufficient data. Other 3 studies were used as qualitative synthesis only. Full details of the selection process were disclosed in Figure 1 and the main characteristics of included studies were displayed in Table 1.

**Table 1:** Summary of included studies evaluating the efficacy or safety of sequential therapies in metastatic renal cell cancer

Study	Year	Race	Design	Median Follow-up	Sequence	Patients, n		Mean age (years)	Male	Clear-cell rate(%)	Median PFS, months			Median OS (months)	NOS score
						(847)	(885)				PFS1	PFS2	PFS		
Sablin et al [11]	2009	French	Retro	N/A	So-Su	68		60	52	82	6.1	6.5	N/A	31.5	8
					Su-So		22	56	18	86	5.1	4.0	N/A	19.1	
Dudek et al [9]	2009	Dutch, American	Retro	86.9/43.9 weeks	So-Su	29		62	22	86	5.1^a^	4.7^a^	18.2^a^	23.8	8
					Su-So		20	58.5	16	80	5.8^a^	2.2^a^	8.6^a^	10.5	
Porta et al [18]	2011	Italian	Retro	N/A	So-Su	90		58	74	84	8.4	7.9	16.3	N/A	8
					Su-So		99	60	67	87	7.8	4.2	12.0	N/A	
Herrmann et al (QL)[17]	2011	German	Retro	N/A	So-Su	54		total	total	Total	N/A	N/A	12.1	28.8	5
					Su-So		33	64	66	72	N/A	N/A	15.4	28.9	
Buchler et al [16]	2012	Czech	Retro	16.7/15.1 months	So-Su	122		60	82	100	N/A	N/A	18.8	30.0	9
					Su-So		138	61	100	100	N/A	N/A	17.7	35.4	
Stenner et al (QL) [12]	2012	Swedish	Retro	N/A	So-Su	10		57.1	N/A	80	5.39	6.01	N/A	N/A	4
					Su-So		11	57.4	N/A	73	12.71	3.71	N/A	N/A	
Calvani et al [14]	2012	Italian	Retro	total 28 months	So-Su	15		70	12	73	6.0	11.0	20.0	Undefined	7
					Su-So		18	61	11	83	7.5	3.0	10.0	27	
Alimohamed et al (QL)	2013 [8]	Canadian, American	Retro	total 36 months	So-Su	152		N/A	N/A	N/A	7.3	5.2	N/A	26.5	4
					Su-So		257	N/A	N/A	N/A	7.6	3.6	N/A	23.0	
Biondani et al [15]	2014	Italian	Retro	66.6/37.1 months	So-Su	125		60	77	38	12	14.1	26.1	35.3	7
					Su-So		104	62	15	14	12	8.0	20.0	27	
Eichelberg et al [13]	2015	German, Australian	RCT	total 10.3 months	So-Su	182		64	139	90	6.9	5.4	12.5	31.5	N/A
					Su-So		183	65	135	84	8.5	2.8	14.9	30.2	

Abbreviations: QL, qualitative synthesis only; Retro, Retrospective; RCT, Randomized controlled trial; N/A, not available; NOS, Newcastle-Ottawa Scale.

^a^ Statas which were described as time to progression, not progression-free survival.

### Efficacy

**Figure 2:**
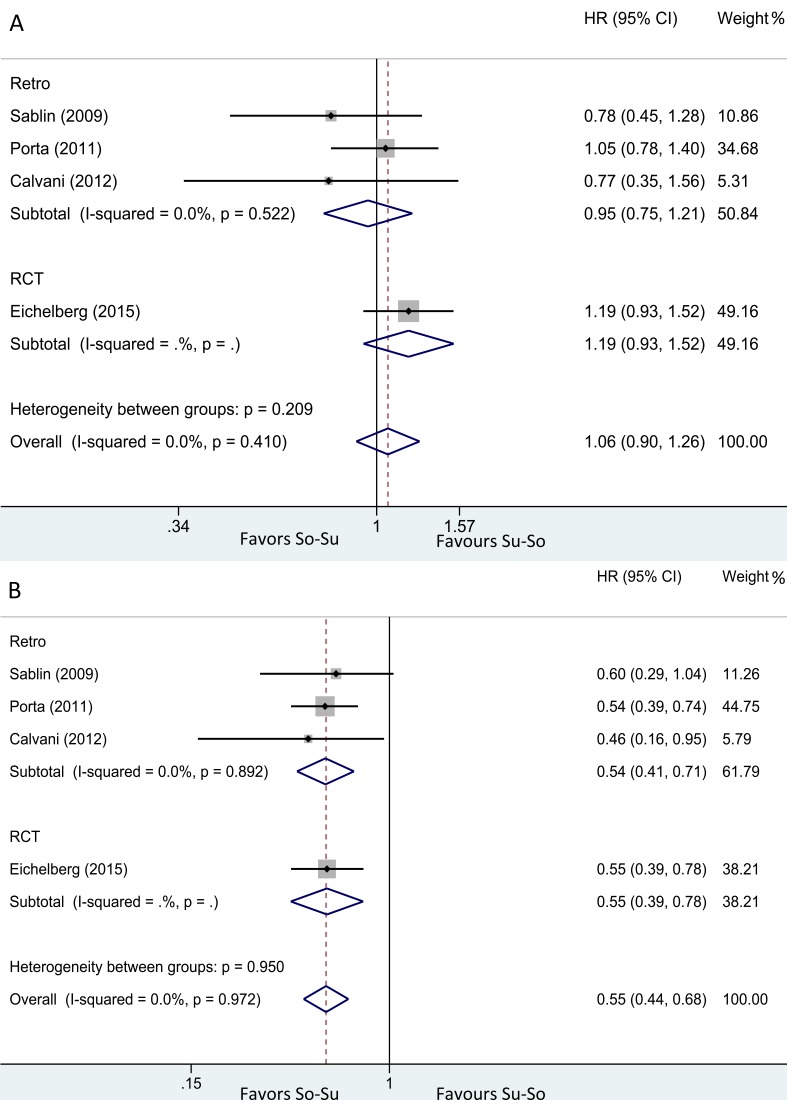
Hazard ratio for (**A**) PFS1, (**B**) PFS2 in overall population treated with So-Su over Su-So.

In a pooled analysis of 3 retrospective studies [8, 11, 18] (So-Su 173 patients, Su-So 139 patients) that assessed PFS1. The cumulative data showed that no significant difference between two groups in PFS1 (fixed effects; HR = 0.95; 95%CI, 0.75-1.21; *p* = 0.702). In RCT [13], 182 received So-Su and 183 received Su-So. PFS1 was similar among two arms (HR = 1.19; 95%CI, 0.93-1.52; log-rank *p* = 0.9). After combined retrospective and RCT, also no significant difference in PFS1 was observed (fixed effects; HR = 1.06; 95%CI, 0.90-1.26; *p* = 0.484) (Figure 2A). All analysis showed no heterogeneity, and the further influence analysis also showed the robustness of our results (Figure 4A).

**Figure 3:**
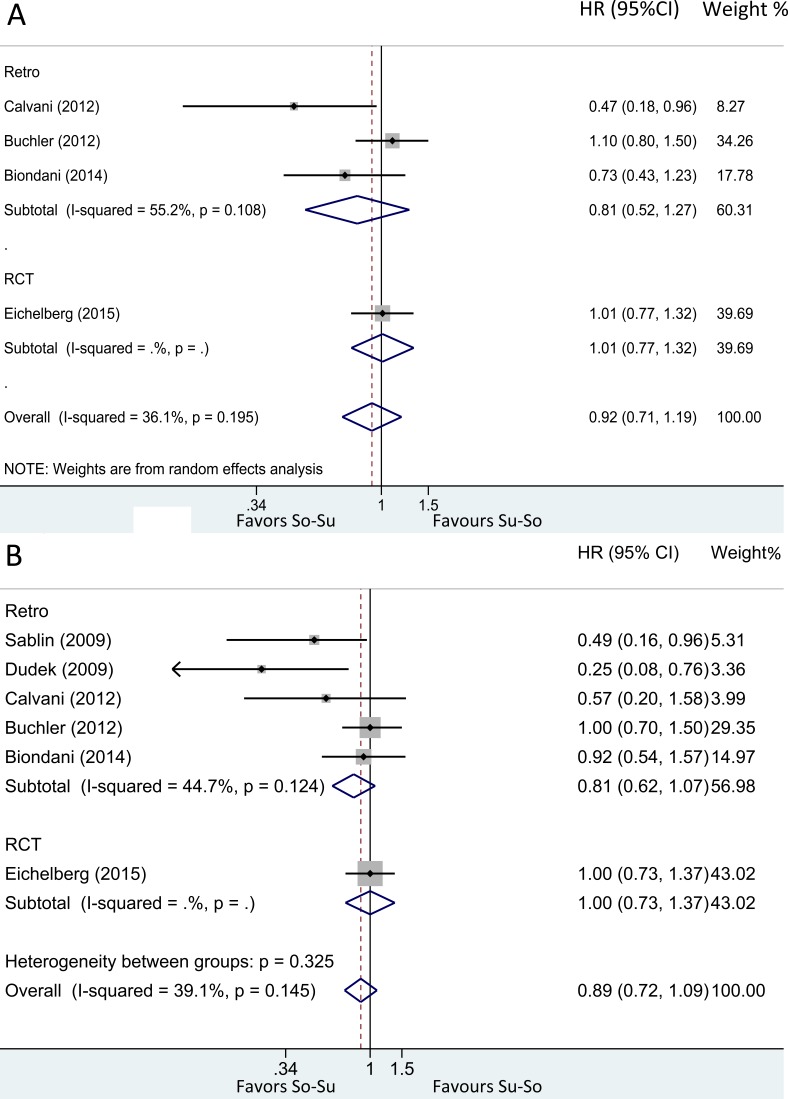
Hazard ratio for (**A**) PFS and (**B**) OS in overall population treated with So-Su over Su-So.

**Figure 4:**
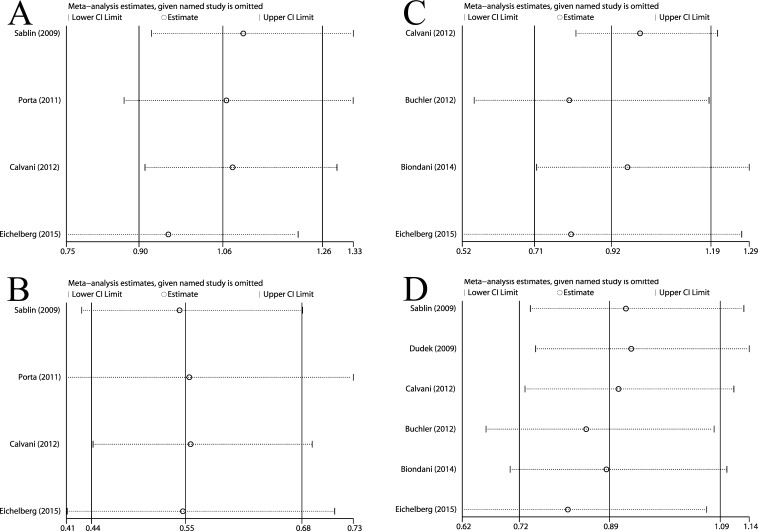
Influence analysis for (**A**) PFS1, (**B**) PFS2, (**C**) PFS and (**D**) OS in overall population treated with So-Su over Su-So.

Pooled analysis of 3 retrospective studies [8, 11, 18] (So-Su 173 patients, Su-So 139 patients) that assessed the PFS2 showed So-Su were more positive in reducing the risk of disease progression in second-line therapy than Su-So (fixed effects; HR = 0.54; 95%CI, 0.41-0.71; *p* = 0.000). In RCT [13] (103 vs 76), median PFS2 was longer for So-Su than for Su-So (HR = 0.55; 95%CI, 0.39-0.78; log-rank *p* < 0.001). After combined all studies, So-Su was still superior to Su-So group (fixed effects; HR = 0.55; 95%CI, 0.44-0.68; *p* = 0.000) (Figure 2B). All analysis showed no heterogeneity, and the further influence analysis also showed the robustness of our results (Figure 4B).

Three studies [8, 15, 16] (262 vs 260) assessed the PFS. The cumulative data of these studies showed no significant difference (random effects; HR = 0.81; 95%CI, 0.52-1.27; *p* = 0.365). In RCT [13] (182 vs 183), So-Su was not superior to Su-So (HR = 1.01; 95%CI, 0.77-1.32; log-rank *p* = 0.5). After combined, still no significant difference in PFS was investigated (random effects; HR = 0.92; 95%CI, 0.71-1.19; *p* = 0.531) (Figure 3A). All analysis showed moderate-to-high heterogeneity, and the further influence analysis also showed the robustness of our results (Figure 4C).

Five studies [8, 9, 11, 15, 16] (359 vs 302) assessed the OS. The cumulative data of these studies showed no significant difference (fixed effects; HR = 0.81; 95%CI, 0.62-1.07; *p* = 0.133). In RCT [13] (182 vs 183), OS was similar in both arms (HR = 1; 95%CI, 0.73-1.37; log-rank *p* = 0.5). After combined, also no significant difference in OS was observed (fixed effects; HR = 0.89; 95%CI, 0.72-1.09; *p* = 0.257) (Figure 3B). All analysis showed moderate-to-high heterogeneity, and the further influence analysis also showed the robustness of our results (Figure 4D).

Additionally, some studies described with median survival, instead of HR and 95%CI, were systemtically reviewed only. Herrmann et al [17] reported 54 patients in So-Su and 33 patients in Su-So group. The median PFS were 12.1 vs 15.4 months and the median OS were 28.8 vs 28.9 months. Stenner et al [12] reported 10 patients in So-Su and 11 patients in Su-So group. The median PFS1 were 5.39 vs 12.71 months and the median OS were 6.01 vs 3.71 months. Alimohamed et al [14] reported 152 in So-Su and 257 in Su-So group. The median PFS1 were 7.3 vs 7.6 months. The median PFS2 were 5.2 vs 3.6 months and the median OS were 26.5 vs 23.0 months.

**Table 2:** Safety overview

Adverse	Eichelberg et al [13]				Buchler et al [16]			
Events, n (%)	First-line So (*n*=177)	Second-line Su (*n*=103)	First-line Su (*n*=176)	Second-line So (*n*=76)	First-line So (*n*=122)	Second-line Su (*n*=122)	First-line Su (*n*=138)	Second-line So (*n*=138)
Any AEs	172 (97)	90 (87)	172(98)	64 (84)	82 (67)^a^	40 (33)^b^	64 (46)^b^	50 (36)^a^
At least one AE	—	—	—	—	92 (75)^c^		88 (64)^c^	
Serious AEs	88 (50)	43 (42)	81 (46)	19 (25)	33 (27)^d^	17 (14)^e^	29 (21)^d^	14 (10)^e^
At least one AE	—	—	—	—	41 (34)^f^		40 (29)^f^	
Grade 3/4 AE	117 (66)	53 (51)	118 (67)	27 (36)	—	—	—	—
AEs related to deaths	12 (6.7)	1 (1.0)	16 (9.1)	2 (2.6)	—	—	—	—

Abbreviations: AE, adverse event; So, sorafenib; Su, sunitinib.

^a^The difference was statistically significant (*P* <0.001).

^b^The difference was statistically significant (*P* = 0.031).

^c^The difference was statistically significant (*P* = 0.045).

^d^The difference was statistically significant (*P* < 0.001).

^e^The difference was not statistically significant (*P* = 0.146).

^f^The difference was not statistically significant (*P* = 0.425).

### Safety

Data for serious adverse events were available from two studies [13, 16]. The most frequently recording adverse events for sorafenib were diarrhea, hand-foot skin reaction. Adverse events for sunitinib were diarrhea, fatigue, hypertension, and nausea. Further toxicity profiles were displayed in Table 2.

**Table 3:** The kinase inhibition profiles of Sorafenib and Sunitinib

Target place	Sorafenib	Sunitinib
Tumor cells	CRAF, BRAF, BRAF V600E, c-KIT, FLT-3	c-KIT, CSF-1R, RET
Vascular endothelium	CRAF, VEGFR-2, VEGFR-3, PDGFR-β	PDGFR-α, PDGFR-β, VEGFR-1, VEGFR-2, VEGFR-3

Abbreviations: CSF-1R, colony stimulating factor receptor; FLT-3, Fms-like tyrosine kinase 3; KIT, stem cell factor receptor; PDGFR, platelet- derived growth factor; RET, glial cell-line derived neurotrophic factor receptor; VEFFR, vascular endothelial growth factor receptor.

## DISCUSSION

In our present study, we investigated whether the therapeutic order of sorafenib and sunitinib in mRCC really mattered. We found that, compared with Su-So, So-Su offers no statistically significant benefit in PFS1, total PFS and OS. PFS2 was statistically longer for So-Su than Su-So. These data may lend support to that So-Su and Su-So provide similar overall clinical benefit in mRCC no matter how to order them.

The approval of targeted therapies as the first-line drug represented a milestone in the treatment landscape for mRCC in the last decade [19]. However, single targeted agent is transient owing to the progressive nature of the disease [20]. Therefore, how to order these agents in an optimal way after disease advanced has become the major focus of the RCC realm. Furthermore, although the NCCN (Version 2.2017) recommends cabozantinib, nivolumab, lenvatinib plus everolimus and Axitinib as a category 1 preferred subsequent therapy option [6], the sunitinib and sorafenib are still essential for mRCC in most of Asian countries, due to differences in approvals in different countries [21]. The optimal sequence has not been determined. There were several clinical trials evaluating sequential targeted therapy in mRCC. RECORD-3, a phase 2 randomized trial, have compared sequential everolimus-sunitinib to sunitinib-everolimus, and suggested that sunitinib-everolimus was a paradigm treatment at progression [22]. Besides, an international phase 3 trial has estimated temsirolimus versus sorafenib as second-line therapy after sunitinib in patients with mRCC [23]. It reported that temsirolimus did not show a benefit compared with sorafenib in PFS, but longer OS observed with sorafenib indicates sequencing sunitinib and sorafenib may benefit patients with mRCC. Consequently, to investigate the optimal sequence for the sorafenib and sunitinib in mRCC may be urgent and significant, at least in most of Asian countries.

Review of the literature, several retrospective and RCT researches have estimated the role of So-Su versus Su-So in patients with mRCC, but with divergent results. Five retrospective studies, which reported by Sablin, Dudek, Porta, Stenner and Calvani et al [8, 9, 11, 12, 18], suggested So-Su was superior to Su-So. However, Herrmann, Buchler, Alimohame, Biondani, Eichelberg et al [13–17] argued that So-Su and Su-So provide similar clinical benefit in mRCC, with no significant differences.

A pooled analysis of this topic was performed by Stenner et al [12] in 2012, but we can discover various drawbacks on it when weighing this article in mind. In that, median combined PFS was 12.1 months on Su-So to 15.4 months on So-Su (95%CI, 1.45-5.12, *p* = 0.0013) among 853 participants. No statistically difference in median PFS1 was noted (median PFS1 was on average 0.62 months longer on So-Su, 95%CI, -1.01 to 2.26, *p* = 0.43). PFS2 was significantly longer in So-Su than Su-So (average increase of 2.66 months, 95%CI, 1.01-4.3, *p* = 0.003). Drawbacks are as follows: First, study inclusion criteria were not clear and lack of a robust quality assessment. Second, there were no assessments of methodological quality, including the evaluation of statistical heterogeneity across the studies. Third, only a few studies were included and they had small sample sizes. Worse still, Dudek et al [9] calculated time to progression rather than progression-free survival, so that cannot be combined with overall PFS1 and PFS2. And they also included four prospective single-arm designed studies which not compared So-Su to Su-So in the analysis. Last but not least, the effect magnitude of this meta-analysis was mean differences in progression-free survival and 95%CI from commencement of both first- and second-line treatment, neither median survival ratio nor hazard ratio. OS was not included as an outcome of this review. All of these methodological flaws in the review made the reliability of this conclusion unclear.

Based on divergent results and defective former meta-analysis, we update this meta-analysis with four new eligible researches. Meanwhile, we estimated HR in more scientific outcomes: PFS1, PFS2, PFS and OS.

Notably, our finally result is consistent with randomized controlled trial SWITCH [13] and the largest retrospective study [14], not former meta-analysis, suggests no cross-resistance between Sorafenib and sunitinib, and both of them are available to mRCC, although with overlapping but not identical kinase inhibition profiles (Table 3). Moreover, one key challenge in this meta-analysis is to interpret the result of PFS2, which was statistically longer for So-Su than Su-So. The reasons for this difference are not clear. According to Eichelberg et al [13] reported more participants received second-line treatment in So-Su than Su-So (57% vs 42%; *p* < 0.01), which may be a reason. Patients receiving sorafenib in first-line are more likely to receive subsequent therapies than those receiving first-line sunitinib [10, 24]. Thus, the result for PFS2 should therefore be interpreted carefully.

The most frequently recording adverse events for sorafenib were diarrhea, hand-foot skin reaction. As for sunitinib, there were diarrhea, fatigue, hypertension, and nausea. All of them were consistent with previous records [25, 26]. Buchler et al [16] reported that overall adverse serious events for sorafenib and sunitinib were statistically lower if the agent was used as the second therapy (*P* = 0.031 for sunitinib and *P* < 0.001 for sorafenib). The rate of serious adverse events was significantly lower for So-Su than vice versa (*P* < 0.001). This indicates cross-tolerance and adaptation were existed between sorafenib and sunitinib probably.

Some limitations of this meta-analysis should be taken into consideration when interpreting the results. This is a meta-analysis based on several original studies rather than individual patient data, although we have tried our best to contact the authors. Second, this analysis is based on retrospective studies and potentially could exist selection and treatment bias due to the retrospective heterogeneous nature. Third, some studies only reported median PFS rather than HR and also cannot be estimated indirectly. Thus, these studies were not synthesized that carry potential publication bias. Forth, the funnel plot which can be used to assess potential publication bias was not performed, due to the small numbers of studies. Finally, subgroup analyses such for race, age, sex, Eastern Cooperative Oncology Group performance status, Memorial Sloan Kettering Cancer Center risk score and other baseline characteristics were not sub-group analyzed, because of the raw data limitation. Further meta-analysis based on individual patient data are expected to deal with this clinical heterogeneity problem.

Given the conclusions of this analysis, sequential therapy with sorafenib and sunitinib is well tolerated and efficient in mRCC. However, there are no evidence supported that sorafenib-sunitinib has the superiority to sunitinib-sorafenib in sequence. The ideal sequence of targeted agents requires further elucidation.

## MATERIALS AND METHODS

### Search strategy

We systematically searched PubMed, Embase, Cochrane Library and ClinicalTrails.gov for relevant studies published between Jan 1, 2006 and Oct 13, 2016. There were no language constraints. Search text and Medical Subject Headings included sorafenib, sunitinib, and metastatic renal cell carcinoma. The complete search strategy used for PubMed was: ((“Carcinoma, Renal Cell”[Mesh] AND metastatic [Title/Abstract]) AND Sorafenib [Title/Abstract]) AND Sunitinib [Title/Abstract]. In addition, we also did a manual search using the reference lists of key literatures and the website of American Society of Clinical Oncology (ASCO).

### Study selection and data extraction

We included eligible studies if they were done in patients with mRCC, definitely compared So-Su to Su-So treatment, and reported the time from start of the first receptor tyrosine kinase inhibitor (rTKI) to the first progression (first-line progression-free survival, PFS1) or the time from start of the second rTKI to second progression or death during second-line therapy (second-line progression-free survival, PFS2) or the time from start of the first rTKI to second progression or death by any cause during second-line therapy (total progression-free survival, PFS) or the time from start of the first rTKI to death by any cause (overall survival, OS), irrespective of the types of studies. Exclusion criteria were considered as follows: single-arm designed research; studies that less than 10 patients in one arm; duplicate publications; studies that were not definitely designed as So-Su and Su-So group, especially for those articles that were designed in an inappropriate or ambiguous way. The outcomes assessed were PFS1, PFS2, PFS and OS.

Eligibility assessments of study tittles and abstracts were performed independently by two reviewers, and studies that potentially complied with predefined eligibility criteria were retrieved for full-text assessment. Finally, certified articles were extracted for further details as follow: the type of studies, follow-up duration, race, total number of participants, So-Su numbers, Su-So numbers, age, sex, clear-cell rate, PFS1, PFS2, combined PFS, OS and the number of participants with serious adverse events. Trials selection and data extraction were analyzed by two contributors with an agreement vale of 95.6%; disagreements between reviewers were resolved by a third expert. Meanwhile, two independent reviewers also assessed risk for bias according to the Newcastle-Ottawa Scale (NOS) [27]. The final NOS assessments were reported as a score between 0 and 9.

### Statistical analysis

We performed this meta-analysis in four major outcomes: PFS1, PFS2, PFS and OS. We analyzed PFS1, PFS2, PFS and OS as time-to-event data; hazard ratio (HR) and 95% confidence intervals (95%CI) acquired directly or indirectly (in accordance with Tierney [28] reported methods) were used to compare results. When meta-analysis was not available, a qualitative synthesis may be done.

We calculated pooled estimates of the HR in PFS1, PFS2, PFS and OS between sequential therapy groups by using random- or fixed- effects model depending on the heterogeneity of the pooled studies. A fixed-effect model (inverse variance method) was applied when substantial heterogeneity not observed. Otherwise, random-effect model (DerSimonian-Laird method) was performed. Account for the differential of study design, we analyzed randomized controlled trials and retrospective studies separately.

Chi-squared Q statistics and I-square testing were used to assess heterogeneity between included trials. The moderate-to-high heterogeneity was considered when *p* values less than 0.1 and I^2^ values greater than 50%. We censored sensitivity by influence analysis, and omitting each study to find potential outliers. Microsoft Office Excel 2013 was used to data collecting. Statistical analysis was calculated by Stata (version 14.0).

This systematic review and meta-analysis is presented in consistent with the Preferred Reporting Items for Systematic Review and Meta-Analysis (PRISMA) Statement [29, 30] and was registered at International Prospective Register of Systematic Reviews (PROSPERO, CRD42016050037).

## Abbreviations

mRCC: metastatic renal cell carcinoma
So-Su: sorafenib followed by sunitinib
Su-So: sunitinib followed by sorafenib
NCCN: National Comprehensive Cancer Network
ASCO: American Society of Clinical Oncology
PFS1: first-line progression-free survival
PFS2: second-line progression-free survival
PFS: total progression-free survival
OS: overall survival
NOS: Newcastle-Ottawa Scale
rTKI: receptor tyrosine kinase inhibitor
HR: hazard ratio
95%CI: 95% confidence intervals
PRISMA: Preferred Reporting Items for Systematic Review and Meta-Analysis
PROSPERO: International Prospective Register of Systematic Reviews
